# Determination of trace elements in placenta by total reflection X-ray fluorescence spectrometry: effects of sampling and sample preparation

**DOI:** 10.1007/s00216-022-04112-5

**Published:** 2022-05-13

**Authors:** Sebastian Hauser, Sophia Andres, Kerstin Leopold

**Affiliations:** 1 Institute of Analytical and Bioanalytical Chemistry, Ulm University, Albert-Einstein-Allee 11, 89081 Ulm, Germany; 2 Universitätsfrauenklinikum Ulm, Prittwitzstrasse 43, 89075 Ulm, Germany

**Keywords:** TXRF, Placenta, Trace elements, Sample preparation and stabilization, Suspension, Fetal and maternal tissue

## Abstract

Placental elemental composition can serve as an indicator for neonatal health. Medical studies aiming at revealing such cause-and-effect relationships or studies monitoring potential environmental influences consist of large sample series to ensure statistically sufficient data. Several analytical techniques have been used to study trace metals in human placenta. However, most techniques require provision of clear liquid sample solutions and therefore time- and reagent-consuming total digestion of biological tissue is necessary. In total reflection X-ray fluorescence spectrometry (TXRF)—a straightforward multielement analytical technique—in contrast suspensions of minute sample amounts can be analyzed directly. Therefore, herein we report on a valid method to prepare homogenous sample suspensions for sustainable and fast TXRF analysis of large sample series. The optimized method requires only 10 mg of powdered placental tissue and 1 mL nitric acid. Suspensions are readily prepared within 30 min and the found mass fractions of major, minor, and trace elements are in good agreement in comparison to analysis of digests. In addition, possible effects on fixation time and the exact sampling location, i.e., maternal vs. fetal side of the placenta, were studied applying this method. Thereby, significant differences for fetal placenta tissue compared to maternal or intermediate tissue were observed revealing accumulation of trace elements in the fetal side of the placenta. Furthermore, considerable depletion of up to 60% mass fraction with longer fixation duration occurred in particular in fetal placenta tissue. These findings help to understand the large ranges of mass fraction of elements in placenta reported in the literature and at the same time indicate the necessity for more systematic investigation of non-homogenous elements distributed in placenta taking sampling and stabilization methods into account.

**Graphical Abstract:**

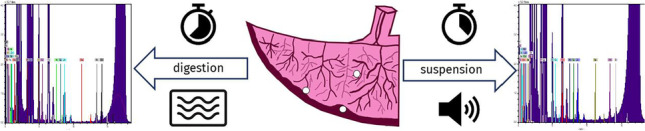

**Supplementary Information:**

The online version contains supplementary material available at 10.1007/s00216-022-04112-5.

## Introduction

The placenta is the organ of remarkable importance in pregnancy and fetal development as well as health of the unborn. Exchange of all nutrients, waste, oxygen, and carbon dioxide between fetus and mother is taking place in the placenta as it is part of the mother’s organism. As the placenta is a temporary formed organ that is simply available after birth, it can be used to study the health status of mother and child of the last 9 months [1, 2]. As part of medical research, various correlations or indications with regard to trace elements have been investigated, as concentrations can be used for example as indicators of neonatal health and birth outcome. Positive correlation between iron, copper, and strontium concentrations in placenta and fetal development in terms of neonatal weight and length as well as placenta weight was found [3, 4], while lower concentrations of chromium, selenium, rubidium, or zinc are found in undersized feti [5]. Furthermore, higher heavy metal concentrations (lead, cadmium) are linked to lower birth weight and length [6].For correct and comprehensive interpretation of such correlations and indications, precise and reliable analytical determination is highly important. However, comparing trace element mass fractions reported in various studies reveals quiet broad concentration ranges [3, 7–9] which are most probably results of both, biological variance and variability of the analytical procedure.

Generally, analytical techniques like atomic absorption spectrometry (AAS) [6, 10, 11], inductively coupled plasma-mass spectrometry (ICP-MS) [12–15], or inductively coupled plasma-atomic emission spectrometry (ICP-AES) have commonly been used for trace element determination in placenta [5, 7, 16, 17]. With the exception of graphite furnace AAS, these techniques require provision of clear liquid sample solutions. This necessitates a digestion step for solid samples, like biological tissue. For sample preparation of biological tissues, typically microwave-assisted digestions using acidic reagent mixtures are applied [5, 7, 12, 18]. The high reagent consumption and limited sample throughput due to constrained device capacities are therefore a bottleneck in the investigation of large sample series. Moreover, since the human placenta is a relatively big organ with individual parts—essentially maternal and fetal side—that show different functions and morphologies, like vascularization [16], sample size (amount) and exact sampling location have to be considered carefully. It is known that placenta tissue is not uniform and best-known example is placental calcification. In this process, deposition of calcium on the basal plate in linear or circular shapes takes place [19]. Calcification mainly depends on gestational age and is found in about 40% at delivery [20]. But also for trace elements like zinc, copper, iron, cadmium, manganese, or nickel, spatial differences were reported [12, 17, 21, 22]. Yet there is no comprehensive understanding for trace element distributions in placenta, because systematic analytical investigations are lacking in this regard. Another difficulty in evaluation of trace elements in placenta reported in literature are differences in the sample stabilization that are likely to cause changes in mass fractions. Chemical fixation by alcohols or aldehydes to preserve the investigated biological tissues is a common procedure—particularly when histological analysis follows [23]. Most widely used chemical fixative is buffered formalin solution. The influence of formalin fixation on leaching of trace metals from biological samples like tissue has been studied with different outcomes. While rapid leaching in significant amounts from rat heart tissue was observed over a period of 6 days, no significant loss was seen in different human organs and tissues after 12 months of fixation [24, 25]. Furthermore, fixation can also increase the concentrations of elements in tissue over time due to contaminations from formalin [26, 27].

In order to address these questions of local distribution and effects resulting from different stabilization of sample tissue, analytical methodologies are required using either micro-sample amounts or providing spatially resolved measurement of tissues. The latter can be achieved using e.g. laser-ablation inductively coupled mass spectrometry (LA-ICP-MS) or micro-X-ray fluorescence spectrometry (μ-XRF), which has been shown for other biological tissues recently [8, 28–30]. However, these techniques require time-consuming sample preparation and stabilization of tissue as well as sophisticated calibration strategies for quantification. Alternatively, total reflection X-ray fluorescence spectrometry (TXRF) offers simple trace element quantification by internal standardization and multielement analysis in minute sample amount. Several approaches show that time- and reagent-effective sample preparation by homogenization and suspension leads to valid trace element quantification in biological tissues by TXRF [31–36]. Thereby, a few microliters of a homogenate are deposited and dried on a sample carrier providing limits of detection (LODs) in the low pg range. Recently, Marguí et al. [19] reported on TXRF analysis of placenta samples using suspensions. Though the authors found significant deviations in the resulting trace element concentrations in comparison to microwave-digested samples, introduction of a correction factor of about 2 led to comparable results. Similarly, however not for biological samples, Bilo et al. [20] applied a correction factor for determination of lead, zinc, and cadmium in suspended soil samples analyzed by TXRF using internal standardization. In both studies, suspensions were prepared using an aqueous emulsifier and the correction factors had to be determined by a reference method, i.e., by digestion prior to measurement by TXRF or ICP-AES. In a previous study performed in our group, we used nitric acid for suspension and partial digestion of mice liver cells and liver tissue and could prove that valid determination of iron and other trace elements in the resulting homogenates by TXRF is possible without applying a correction factor [37]. Accordingly, the aim of this work was the development of a fast and simple sample preparation technique for TXRF multielement analysis in human placenta to study the influence of sample location as well as sample fixation time on trace element mass fractions.

## Materials and methods

### Cleaning procedures

#### Sample carriers

Quartz glass sample carriers were first wiped using cleanroom wipes (Spec-Wipe 3, VWR International LLC, Radnor, PA, USA) soaked with ultrapure water (UPW; 18.2 MΩ cm, 100 μS cm^−1^, arium pro, Sartorius AG, Göttingen, Germany) and then placed for 2 h in 10% Hellmanex III solution (Hellma Analytics GmbH & Co KG, Mühlheim, Germany) at 80 °C. Thereafter, they were rinsed three times with UPW and placed in a 10% nitric acid bath for 2 h at 80 °C. Finally, after rinsing three times with UPW again, the carriers were placed in a drying oven for 2 h at 120 °C. Cooled carriers were coated with 10 μL silicone solution (in 2-propanol, Serva electrophoresis GmbH, Heidelberg, Germany) and checked for purity by TXRF measurement (measurement time 100 s, 50 kV, 600 μA) prior to use.

#### Consumables

All consumables like tips, tubes, flasks, and funnels were cleaned for at least 24 h in 10% nitric acid baths and stored until use in 0.5% nitric acid. Prior to use, each item was rinsed three times with UPW. Agate mortar and ball mill were rinsed three times with UPW followed by 5% nitric acid and UPW and dried at 60 °C. Microwave vessels were cleaned after every use by running a cleaning microwave program following the same procedure and chemicals as for the digests.

### Sample collection and homogenization

The study design was approved and conducted in compliance with the ethics guidelines of Ulm University. After education and the mother’s consent, the placenta is retrieved during a planned caesarean section after ≥ 37^th^ week of pregnancy and placed in formalin subsequently. After fixation in formalin, single cotyledons from three different locations were cut out using a diamond scalpel and placed in Petri dishes. Partition into individual samples classified into (a) maternal side (decidua, pointing towards the uterus), (b) fetal side (top half of the placenta, side of the umbilical cord), and (c) intersection of fetal and maternal side (longitudinal cut; intermediate between chorionic villi and decidua) was done macroscopically. The placenta pieces were dried to mass constant—resulting in weights between 0.5 and 1.5 g—and crushed in an agate mortar into smaller pieces. These were placed in a planet ball mill (S1, Retsch GmbH, Haan, Germany) for 30 min at a speed of 80 rpm. These two steps were repeated and finally all remaining flakes or particles were crushed by mortar and pestle until a fine homogenous powder was achieved which was stored in precleaned Eppendorf tubes at room temperature.

### Sample preparation for TXRF analysis

#### Digestion

For microwave digestion (Multiwave 3000, Anton Paar, Ostfildern, Germany) based on US Environmental Protection Agency (EPA) method 3052 [38], approx. 250 mg of placenta powder was weighed into 80-mL Teflon microwave vessels and 8 mL of nitric acid (65% Normapur, VWR International LLC, Radnor, PA, USA) and 2 mL of H_2_O_2_ (30%, aqueous solution, p.A., Merck KGaA, Darmstadt, Germany) were added. Subboiled nitric acid was used in all following steps (DST-1000, Savillex Corporation Eden Prairie, MN, USA). Digestion took place 10 min at power of 1000 W followed by a 30-min cooling step. During digestion, a temperature between 170 and 200 °C and a pressure of about 30 bar were reached. After cooling down to room temperature, the solutions were transferred into 25-mL volumetric flasks and diluted to the mark with UPW. One thousand microliters of this solution was added to 50 μL of vanadium standard solution (100 mg L^−1^, in 3% w/w HNO_3_ Suprapur, Merck KGaA, Darmstadt, Germany) and homogenized for 60 s at 2500 rpm (Digital mini vortex mixer, VWR International LLC, Radnor, PA, USA). Then, 10 μL was applied to a quartz glass carrier (30 × 3 mm, BrukerNano GmbH, Berlin, Germany) and the solvent was evaporated on a heating plate (VMS-C7, VWR International LLC, Radnor, PA, USA) at 60 °C for 90 min. Triplicates were prepared for each sample. Sample stock solutions were stored at 4 °C in the dark. All solutions and liquids were vortexed for 30 s at 2500 rpm before withdrawal of volume. All steps were carried out under laminar flow box (SuSi Super Silent, Spectec GmbH, Erding, Germany). UPW was used for all dilution steps.

#### Acidic suspension

For preparation of acidic sample suspensions, approx. 10 mg of fine placenta powder was weighed into precleaned 2-mL Eppendorf tubes and subsequently 50 μL of vanadium standard solution (100 mg L^−1^) and 1000 μL of nitric acid were added. Suspensions were vortexed for 30 s at 2500 rpm and placed in an ultrasonic bath at 40 °C for 30 min. Shortly before applying an aliquot of the acidic suspension onto a TXRF sample carrier, suspensions were vigorously shaken again for 30 s at 2500 rpm.

### Instrumentation, calibration, and validation

For TXRF measurements, the following method parameters and instrumentation were used. S2 Picofox (BrukerNano GmbH, Berlin, Germany) equipped with a molybdenum X-ray tube operated at 50-kV voltage and 600-μA current was used. Software used for measurements and evaluation was Spectra 7.8.2.0 (BrukerNano GmbH, Berlin, Germany). Background calculation was carried out with 40 cycles based on escape peaks and an optimized profile Bayes fit with a step width of 1 and a maximum number of stripping cycles of 100 for deconvolution. Measurement time was set to 500-s live time. Measurements were carried out under nitrogen atmosphere (5.0, MTI, Neu-Ulm, Germany). Vanadium standard solution used as internal standard (IS) for TXRF measurements was prepared from 1000 mg L^−1^ vanadium standard by dilution in 3% nitric acid. Vanadium was chosen as IS as no peak is present at 4.95 keV (V Ka) or in proximity in digested sample. Validity of IS was double-checked by calibration against 1000 mg L^−1^ gallium and nickel standard solution. In each sample digestion and suspension run, one blank solution was included to check for contaminations. Quantitative evaluation was achieved using the IS and correlating its concentration with respect to the relative sensitivities to the net intensities found for the analyte and the IS as given by the following equation:

1 $${c}_A=\frac{N_A}{S_A}\ast \frac{S_{IS}}{N_{IS}}\ast {c}_{IS}$$

with net intensity *N*_*i*_, relative sensitivity *S*_*i*_, and concentration *c*_*i*_with indices *IS* for internal standard and *A* for analyte.

Limits of detection and quantification were calculated from spectra with lowest analyte amounts by the following equations.

2 $$\mathrm{LOD}=\frac{3\ {c}_i\sqrt{N_{BG}}}{N_i}$$

3 $$\mathrm{LOQ}=\frac{10\ {c}_i\sqrt{N_{BG}}}{N_i}$$

with background intensity *N*_*BG*_ and peak intensity *N*_*i*_.

Only elements fulfilling the LOQ criterion were included in the quantitative evaluation. Grubb’s outlier test was used to identify and exclude outliers. Comparison between two datasets was done by Welch’s *t*-test. The number of quantifications fulfilling this criterion is given by *n*, while *N* refers to the number of individual number of digests or suspensions for each individual subsample/aliquot.

## Results and discussion

### Method optimization and validation

**Fig. 1:**
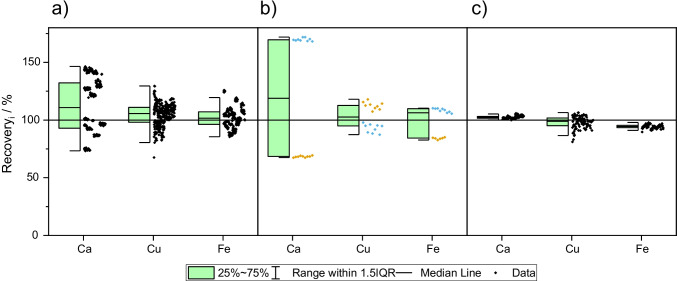
Investigation of sample homogeneity by evaluation of calcium, copper, and iron recovery by TXRF determination in suspensions compared to digestions after (**a**) simple grinding procedure and random sampling; (**b**) simple grinding procedure and subsampling of fine or coarse sample substance; and (**c**) enhanced grinding procedure and random sampling. *N* = 10, Box plot presentation gives median, 25–75% percentiles and includes all data points for each element. Black diamonds: data points from random sampling (*n* = 30); blue diamonds: data points from fine powder sampling (*n*_sub_ = 10); orange diamonds: data points from coarse powder sampling (*n*_sub_ = 10)

First, a straightforward sample preparation method for TXRF analysis of large sample series of small amounts of human placenta was developed. In preliminary experiments, we could confirm that suspending placenta sample in aqueous emulsifier solution (Triton® X-100) results in minor recoveries compared to analysis of digested sample, as reported recently by Marguí et al. [19]. Accordingly, in this work, preparation of acidic suspensions of placenta samples using nitric acid was tested and optimized in order to improve recovery rates. First, sample amounts, homogenization, and ultrasonic duration were varied until optimized conditions for suspending placenta in nitric acid were found. One crucial aspect here was grinding the material to a fine powder in order to obtain reproducible results for replicate analysis of only 10 mg of the sample. Figure 1 shows calcium, copper, and iron recovery by TXRF determination in suspensions after different grinding procedures. It is evident that a simple grinding procedure of 30 min in a ball mill yields insufficient homogeneity of the material, as observed not only by high variation of results from replicate sample aliquots (Fig. 1a), but which is even more evident when using subsamples of fine or course material, respectively, as shown in Fig. 1b. In the enhanced grinding procedure, the process is repeated which leads to significantly lower variation in the replicate investigation of 10-mg aliquots (see Fig. 1c). Next, the influence of variation of ultrasonic treatment duration was investigated. Samples were placed for 15 min, 30 min, or 45 min, respectively, in an ultrasonic bath at 40 °C. The results reveal comparable recoveries for most elements (see Table 1 and SI Fig S1); however, for the elements detected close to their low limit of detection, the percentage of quantification events increases with higher duration of ultra-sonification. Therefore, as a compromise, 30 min for ultrasonic treatment was chosen in all following experiments. Finally, optimized sample preparation consists of a 2-fold grinding of the dried material followed by taking a 10-mg sample, addition of internal standard and nitric acid, and homogenization by ultrasonic treatment for 30 min at 40 °C. This procedure is similar to the approach for TXRF investigation of liver tissues as developed in a previous study of the authors [37]. Here, successful recovery of copper, iron, manganese, and zinc in the standard reference material 1577c bovine liver was already shown. Similarly, placenta tissue suspended in nitric acid shows a high degree of digestion resulting in suspensions that are almost clear. In comparison to aqueous emulsions in which the sample is still present in the form of fine powder particles, here less matrix effects occur and sample aliquots are more homogenous resulting in higher recoveries.

**Table 1:** Mass fractions after sonification for 15, 30, and 45 min, respectively, given as mean ± 1 SD with number of detections (*n*)

Sonification duration		15 min		30 min		45 min	
Element		w/μg g^−1^	*n*	w/μg g^−1^	*n*	w/μg g^−1^	*n*
19	K	6022 ± 125	27	7208 ± 452	90	6921 ± 414	27
20	Ca	28,075 ± 378	27	28,139 ± 327	90	28,217 ± 283	27
26	Fe	487 ± 25	27	469 ± 8	90	481 ± 6	27
28	Cu	5.35 ± 0.24	27	5.31 ± 0.28	90	5.31 ± 0.23	27
30	Zn	64.6 ± 0.9	27	69.1 ± 1.8	90	67 ± 1.7	27
37	Rb	5.8 ± 0.15	27	7.05 ± 0.57	90	6.78 ± 0.56	27
38	Sr	1.91 ± 0.56	8	2.14 ± 0.41	52	1.64 ± 0.28	18

**Fig. 2:**
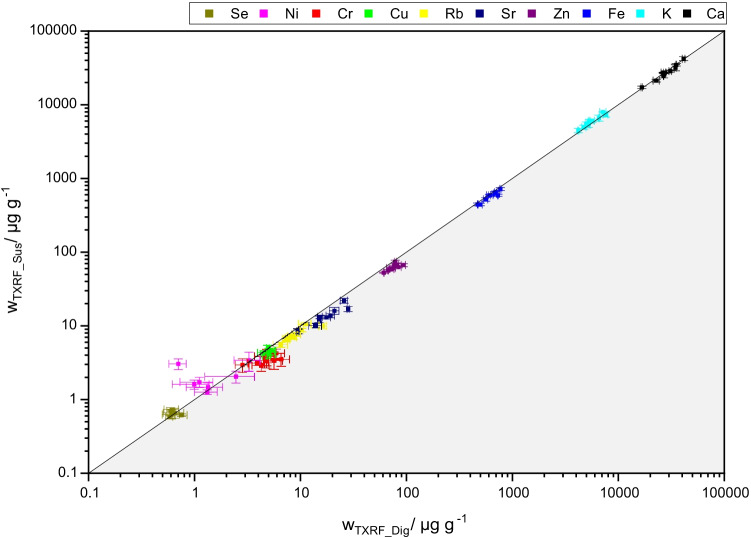
Comparison of results of element determination by TXRF in digests and suspensions. Solid black line indicates bisector. Results are expressed as mean ± SD with *n* > 3, *N* = 9

Nevertheless, as the matrix is somehow different, further validation and investigation of additional elements were achieved in this study. First, validity of the results has been studied by investigation of Zn, Fe, and Ca in digests by ICP-AES as a reference method. In comparison to TXRF measurement in digests, the obtained recovery rates were 102 ± 13% (Ca), 112 ± 5% (Fe), and 120 ± 11% (Zn) which proves comparability of the two detection techniques. Moreover, the results for all quantifiable elements in acidic suspensions versus in digests as measured by TXRF were compared as shown in Fig. 2.

Values range from sub-μg g^−1^ (Se) to several tens of mg g^−1^ (Ca). As can be seen, a good agreement between the values found in suspensions and in digests measured by TXRF is obtained revealing that the use of nitric acid instead of an aqueous emulsifier solution allows direct quantification without a correction factor also for the matrix placenta. In contrast to our study on liver tissue [37], in placenta correct and non-interfered determination of Ca was clearly possible (Ca recoveries: 92–103%) as well as quantification of the trace element Se (recoveries: 93–117%). The only exception was the element Ni, which was found in concentrations close to the quantification limit and significant overestimations in the suspensions occurred that may result from an unknown source of trace contamination in the preparation of the suspensions or from overlap of Kα lines of iron which is present at much higher mass fractions. Generally, as to be expected, correlations between mass fractions from digested and suspended samples are less consistent and data spreads more for elements with lower mass fraction compared to higher mass fraction. All mass fraction and recovery values can be found in the SI in Tables S1 to S9. Interpretation of this data, however, has to take into account that the observed inhomogeneity of the matrix adds to the deviations observed when comparing two subsamples. This point will be discussed further in section “Effects of sampling”.

### Analytical figures of merit

Measurement precision by TXRF in suspension is similar to that in digest with relative standard deviations (RSDs) below 10%, except for Ni and Se, which were both detected either close to their limit of quantification (LOQ) or below. Hence, sufficient homogeneity of the applied aliquot and the internal standard is given. The limits of detection (LODs) are—as to be expected—slightly lower after digestion, but the differences are more or less neglectable (see Table 2). Further comparison between both sample preparation techniques is given in the following Table 3. It is obvious that ultrasonic suspension offers significantly increased sample throughput compared to microwave-assisted digestion. A higher number of samples can be prepared per time as a significant higher number of samples can be prepared simultaneously. From an effort point of view, it can be said that microwave-assisted digestion is much more elaborate compared to suspension. Even though the preparation steps are similar, transfer and dilution steps are required after digestion, while this is not necessary in case of suspension. The most important difference is however the 25-times lower sample consumption for suspension method. This allows investigation of defined locations in placenta in future studies and will make monitoring of potential effects that occur across maternal and fetal side of the placenta possible.

**Table 2:** Relative standard deviations and limits of detection for TXRF determination in suspensions compared to in digests (*n*_Sus_ > 31, *n*_Dig_ > 38)

Atomic number	Element	RSD_Sus_ / %	RSD_Dig_ / %	LOD_Sus_ / pg	LOD_Dig_ / pg	$\frac{\mathrm{LO}{\mathrm{D}}_{\mathrm{Sus}}}{\mathrm{LO}{\mathrm{D}}_{\mathrm{D}\mathrm{ig}}}$
19	K	6.6	3.6	543	415	1.31
20	Ca	4.6	2.9	262	191	1.37
24	Cr	20.0	16.5	77.7	56.9	1.37
26	Fe	4.2	2.9	48.7	34.1	1.43
28	Cu	6.9	7.2	25.9	17.5	1.48
28	Ni	16.5	30.1	31.5	21.5	1.47
30	Zn	3.3	4.0	22.4	15.2	1.47
34	Se	8.3	14.2	15.9	9.7	1.64
37	Rb	6.7	8.4	18.3	11.8	1.55
38	Sr	7.6	7.1	22.3	13.6	1.64

**Table 3:** Comparison of methodical parameters for sample preparation by microwave-assisted digestion versus ultrasonic-assisted acidic suspension

Parameter	Microwave-assisted digestion	Ultrasonic-assisted acidic suspension
Reagent consumption	8 mL nitric acid 2 mL hydrogen peroxide	1 mL nitric acid
Sample weight	250 mg	10 mg
Duration of sample preparation	5-min heating 10-min digestion 25-min cooling	30-min ultrasonication
Sample throughput	Limited by microwave rotor capacity	Limited by size of ultrasonic bath
Post-processing	Transfer to flask and dilution	-

### Effects of sampling

**Fig. 3:**
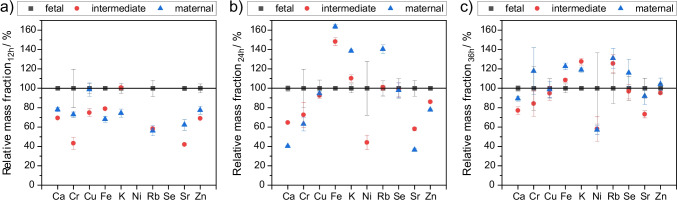
Effect of sampling location on mass fractions after 12 h (**a**), 24 h (**b**), and 36 h (**c**) of fixation time. Results are expressed as relative mass fractions (fetal, black square; intermediate, black circle; maternal, black triangle) calculated from mean *w*_Fetal_ as reference. Error bars represent ± 1 SD with *n* > 3; *N* = 1. Values for mass fractions and number of detections *n* are given in the SI Tables S1-9

Notably, the observed concentration ranges for the individual elements are quite large (see Fig. 2). This is a result of the different sampling locations (fetal, maternal, intermediate) and fixation durations (12, 24, 36 h) of the cut-out samples. As can be seen in Fig. 3a, higher mass fractions in fetal tissue are found after 12-h fixation. Consistently, the intermediate sections show average values between fetal and maternal side. Such spatial mass fraction differences are rarely reported and published results are not consistent. While higher Zn mass fractions in maternal tissue were reported by Manci and Ronco [21, 22], Roverso et al. [12] found a higher mass fraction in fetal tissue. Osman et al. [17] reported on deviations of up to 50% in mass fraction of different placenta parts without indicating further information on location. For Ca, an increase of 39% towards the periphery was observed, whereas for Fe, a depletion by 22% was observed [21]. No differences were found in one study for Cu [22], while others report changes by 200% [17] or significant depletion towards periphery [39].

**Fig. 4:**
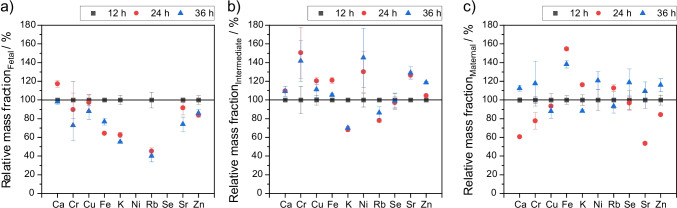
Effect of fixation time on mass fractions for fetal (**a**), intermediate (**b**), and maternal (**c**) tissue. Results are expressed as relative mass fractions (12 h, black square; 24 h, black circle; 36 h, black triangle) calculated from mean *w*_12h_ as reference. Error bars represent ± 1 SD with *n* > 3; *N* = 1. Values for mass fractions and number of detections *n* are given in the SI Tables S1-9

After longer fixation times, the relative mass fractions change—as can be seen in Fig. 3b and c —suggesting leaching of elements. Trace elements seem to deplete slightly in fetal placenta with longer fixation times (see Fig. 4a), whereas no such trend is obvious in the maternal part and intermediate (see Fig. 4b and c). A possible explanation might be the reversal of natural process. The fetal part consists of chorionic villi, which have a large surface. These villi absorb nutrients and trace metals from the maternal blood in the intervillous space. During fixation in formalin, the reverse process may occur, leading to leaching placenta ingredients into formalin. The large surface of the fetal tissue may facilitate the leaching process into the fixation solution, as also found by Sato et al. [40]. Significance and share of decreasing mass fractions shrink with an increasing fixation time. The degree of leaching ranges from 15% (zinc) up to 60% for Rb with the exception of calcium (98%). These findings are in good agreement with literature data [41, 42] where the strongest depletion was found for rubidium and potassium, and mediocre leaching for zinc and iron. It indicates either strong interaction with fixation solution or weak interaction with placental tissue, possibly due to the fact that Rb and K ions are weak acids as they both have high ion radii and are only single charged. In conclusion, different leaching degrees occur in different types of placenta tissue—especially for fetal tissue leaching is significant.

Apparently, the above-described effects add to biological variability and may be the reason for the very broad mass fraction ranges reported in human placenta in literature (see Table 4). Hence, it is obvious that sampling and sample preparation are crucial in trace element determination in human placenta and more consistent methods, as well as investigation of larger sample series, are required in order to provide comparable results that allow meaningful interpretation of the data.

**Table 4:** Determined mass fraction ranges in this work and comparison to literature data

	This work^a^			Values from literature	
Element	Median	0.25^th^	0.75^th^	Mean value or range	Ref.
Ca	27,901.7	24,890.8	34,879.0	5500 ± 200 ^a^	[19]
				3766 ± 330 ^c^	[43]
				12,600 ± 2,220* ^a^ 17,400 ± 2,940* ^a^	[18]
				100–388,236* ^a^	[7]
				665–16,537* ^d^	[44]
				600–32,000 ^d, mat^ 700–76,000 ^d, fet^	[8]
K	5357.1	4948.3	6623.1	5300 ± 30 ^a^	[19]
				9681 ± 2196 ^c^	[43]
Fe	605.6	527.9	719.3	615* ^a^	[7]
				564 ± 5 ^a^	[19]
				719.82 ± 228.78 ^d^	[9]
				630 ± 150 ^d^	[45]
				600 ± 90 ^d^	[19]
Zn	75.9	67.0	80.6	49 ± 1 ^a^	[19]
				72–82.8 ^a^*	[7]
				50–60 ^c^	[43]
				59.6 ± 14.2 ^d^	[45]
				51 ± 2 ^d^	[19]
Sr	16.8	14.6	22.5	0.155–16.990 ^d^	[3]
				1.7 ± 0.6 ^c^ 2.9 ± 0.6 ^c^	[43]
				0.600 * ^b^	[13]
				1.0–8.4 ^b^	[12]
				12–12.2 * ^a^	[18]
Rb	8.6	7.6	10.0	8.2 ± 0.4 ^a^	[19]
				15.3 ± 3 ^c^	[43]
Cu	5.0	4.5	5.2	5.59 ± 0.03 ^a^	[19]
				6.4–8.0 ^c^	[43]
				3.6–16.8* ^a^	[7]
Cr	4.7	3.7	5.7	9.6–12.6* ^a^	[18]
				0.13 ^b^	[12]
				0.60* ^b^	[13]
Ni	1.7	1.3	2.7	0.560 * ^b^	[13]
				0.235 ± 0.138 * ^d^	[44]
				5.8 ^b, fet^ 1.0 ^b, mat^	[12]
Se	0.6	0.6	0.7	0.8 ± 0.4–1.1 ± 03 ^c^	[43]
				0.840* ^b^	[13]
				0.63 ^b, fet^ 0.65 ^b, mat^	[12]

Annotations: All mass fractions given in μg g^−1^

*Calculated values from wet weight by factor 6

^a^TXRF, ^b^ICP-MS, ^c^EDXRF, ^d^ICP-AES, ^mat^maternal tissue, ^fet^fetal tissue

## Conclusion

In this work, a fast and simple sample preparation methodology for multielement analysis of placenta with TXRF using internal standardization as quantification method without a correction factor was achieved. For major elements like potassium, calcium, and iron, a very good agreement between measurements in digests *versus* in suspensions was found. For minor and trace elements, the found values spread more, but still the observed trends are clearly present and the recoveries are acceptable. Found LODs and RSDs are comparable to those of digestion method and indicate agreeable method performance. In comparison to established microwave digestion, ultrasonic suspension is simpler and faster and a higher sample throughput can be achieved. Here, the influence and importance of sampling parameters and sample preparation in determination of trace elements in placental tissue was shown in this study. Elemental distribution in human placenta tissue is not homogenous, but differences between fetal and maternal placenta sides and their corresponding tissue type (intervillous vs. decidual) are present. For example, calcium and strontium mass fractions are substantially higher for fetal placenta tissue compared to maternal tissue. Moreover, it could be proven that accumulation of trace elements in the fetal side of the placenta occurs. On the other hand, the comparison of literature data for mass fractions in human placenta covers very broad ranges. Obviously, methodological variability and biological variability add to these differences which strongly indicates the need to systematically acquire spatially resolved data. In this regard, application of elemental mapping techniques in future studies seems meaningful and the herein developed and validated TXRF method for direct investigation of minute samples can serve as a tool to validate such methods that require fixation prior to preparation of cross sections. In this context, also the observed influence of different fixation times in formalin is an important finding. Significant depletion of mass fraction is present in fetal placenta tissue, e.g., mass fractions of rubidium and potassium decline to 40% and 60%, respectively, after 36 h in formalin in comparison to a fixation time of only 12 h. Hence, the benefits of low sample amounts and multielement TXRF analysis in combination with the herein presented sample preparation method allow high sample throughput and more localized investigation of placenta in future studies in order to investigate more systematically element distribution in placenta. Thus, the present work can be seen as a first step towards a systematic data acquisition.

## Supplementary Information

Below is the link to the electronic supplementary material.

Supplementary file1 (PDF 187 kb)
