# Supplementary material for: Determination of trace elements in placenta by total reflection X-ray fluorescence spectrometry: effects of sampling and sample preparation
Source: Anal Bioanal Chem. 2022 May 13;414(15):4519–29. doi: 10.1007/s00216-022-04112-5 (PMC9142463; doi:10.1007/s00216-022-04112-5)
Supplement: Supplementary file 1 — Supplementary file1 (PDF 187 kb) [file 216_2022_4112_MOESM1_ESM.pdf]

SUPPORTING INFORMATION

Determination of trace elements in placenta by total reflection X-ray fluorescence spectrometry: Effects of sampling and sample preparation

Sebastian Hauser[1], Sophia Andres[2], Kerstin Leopold[1*]

[1] Institute of Analytical and Bioanalytical Chemistry, Ulm University, Albert-Einstein-Allee 11, 89081 Ulm, Germany

[2] Universitätsfrauenklinikum Ulm, Prittwitzstrasse 43, 89075 Ulm, Germany

SH orcid 0000-0002-4769-6266

SA orcid 0000-0001-7828-2501

* KL orcid 0000-0003-0586-7239 kerstin.leopold@uni-ulm.de

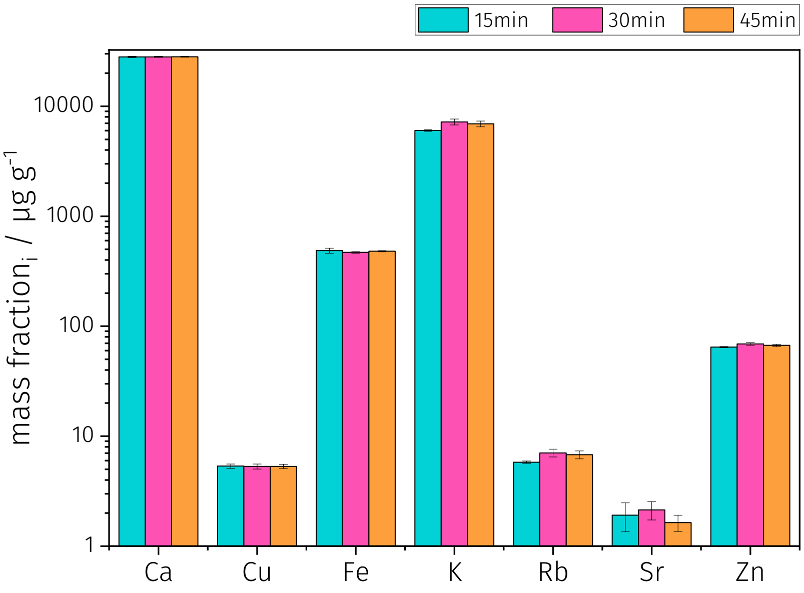

Figure S1: Investigation of duration of ultrasonic treatment. (N=5, 30 min: n=90, exc. n=52 (Sr); 15 min: n=27, exc. n=8 (Sr); 45 min: n=27, exc. n=18 (Sr); mean ± SD.)

Table S1 Determined mass fractions *w* and number of quantifications *n* in fetal tissue after a fixation time of 12 h after digestion (Dig) and suspension (Sus). Results are expressed as mean ± SD with N = 4.

Element	w_{Dig} / µg g^{-1}	*n*	w_{Sus} / µg g^{-1}	*n*	recovery / %

Ca	35,179 ± 238	9	34,905 ± 1,394	27	99.2 ± 4.0
K	7,567 ± 395	9	7,387 ± 580	27	97.6 ± 9.3
Fe	729 ± 6	9	590 ± 20	27	80.9 ± 2.9
Zn	93.4 ± 4.3	9	66.8 ± 3.1	27	71.5 ± 5.7
Sr	28.1 ± 0.7	9	17 ± 1.3	27	60.5 ± 5.3
Rb	16.23 ± 1.37	9	9.99 ± 0.88	27	61.6 ± 10.0
Cu	5.47 ± 0.3	9	4.15 ± 0.25	27	75.8 ± 7.2
Cr	6.55 ± 1.3	7	3.52 ± 0.69	16	53.8 ± 22.4
Se	<LOQ	0	0.616 ± 0.026	9	---
Ni	<LOQ	0	3.058 ± 0.509	27	---

Table S2 Determined mass fractions *w* and number of quantifications *n* in intermediate tissue after a fixation time of 12 h after digestion (Dig) and suspension (Sus). Results are expressed as mean ± SD with N = 4.

Element	w_{Dig} / $\mu g\ g^{-1}$	*n*	w_{Sus} / $\mu g\ g^{-1}$	*n*	recovery / %
Ca	22,775 ± 1,497	9	21,270 ± 955	27	93.4 ± 7.8
K	7,141 ± 485	9	7,952 ± 156	27	111.4 ± 7.1
Fe	612 ± 44	9	592 ± 20	27	96.7 ± 7.9
Zn	71.5 ± 6.7	9	60.6 ± 1.8	27	84.8 ± 9.7
Sr	13.9 ± 1.9	9	10.1 ± 0.7	27	72.7 ± 14.6
Rb	11.09 ± 1.51	9	10.58 ± 0.37	27	95.4 ± 14.0
Cu	4.1 ± 0.22	9	4.34 ± 0.26	27	105.9 ± 8.4
Cr	2.83 ± 0.42	4	2.95 ± 0.63	5	104.4 ± 26.6
Se	0.599 ± 0.046	9	0.626 ± 0.082	8	104.6 ± 15.6
Ni	1.104 ± 0.082	3	<LOQ	-	---

Table S3 Determined mass fractions *w* and number of quantifications *n* in maternal tissue after a fixation time of 12 h after digestion (Dig) and suspension (Sus). Results are expressed as mean ± SD with N = 4.

Element	w_{Dig} / $\mu g\ g^{-1}$	*n*	w_{Sus} / $\mu g\ g^{-1}$	*n*	recovery / %
Ca	27,456 ± 704	9	27,256 ± 734	27	99.3 ± 3.7
K	5,637 ± 314	9	6,079 ± 322	27	107.8 ± 8.0

Fe	497 ± 23	9	444 ± 17	27	89.3 ± 5.8
Zn	72.4 ± 3.8	9	59.1 ± 1.5	27	81.6 ± 4.4
Sr	17.6 ± 1.5	9	13.1 ± 0.7	27	74.4 ± 9.4
Rb	9.12 ± 0.85	9	7.7 ± 0.5	27	84.4 ± 10.8
Cu	5.41 ± 0.37	9	4.66 ± 0.22	27	86.1 ± 8
Cr	<LOQ	0	3.32 ± 0.63	8	---
Se	0.599 ± 0.059	4	<LOQ	-	---
Ni	1.303 ± 0.143	5	1.613 ± 0.228	14	123.8 ± 20.6

Table S4 Determined mass fractions *w* and number of quantifications *n* in fetal tissue after a fixation time of 24 h after digestion (Dig) and suspension (Sus). Results are expressed as mean ± SD with N = 4.

Element	w_{Dig} / *μg g*$^{-1}$	*n*	w_{Sus} / *μg g*$^{-1}$	*n*	recovery / %
Ca	41,297 ± 1243	9	42,113 ± 2609	27	102.0 ± 7.0
K	4,726 ± 216	9	4,941 ± 333	27	104.5 ±8.4
Fe	469 ± 3	9	445 ± 19	27	94.9 ±4.1
Zn	78.3 ± 1.6	9	74.2 ± 3.7	27	94.8 ± 5.1
Sr	25.8 ± 2.1	9	22 ± 1.7	27	85.3 ± 10.5
Rb	7.33 ± 0.57	9	6.41 ± 0.35	27	87.4 ± 9.1
Cu	5.33 ± 0.46	9	4.6 ± 0.38	27	86.4 ± 11.2
Cr	5.89 ± 1.16	8	4.26 ± 0.62	17	72.4 ± 22.3
Se	0.591 ± 0.059	4	<LOQ	-	---
Ni	3.26 ± 0.901	9	3.411 ± 0.989	27	104.6 ± 41

Table S5 Determined mass fractions *w* and number of quantifications *n* in intermediate tissue after a fixation time of 24 h after digestion (Dig) and suspension (Sus). Results are expressed as mean ± SD with N = 4.

Element	w_{Dig} / *μg g*$^{-1}$	*n*	w_{Sus} / *μg g*$^{-1}$	*n*	recovery / %
Ca	26,679 ± 165	9	24,678 ± 1,624	24	92.5 ± 6.1
K	5,208 ± 176	9	5,380 ± 469	24	103.3 ± 9.6
Fe	696 ± 19	9	649 ± 27	24	93.2 ± 4.7
Zn	67.4 ± 0.6	9	56.6 ± 1.4	24	84.0 ± 2.3

Sr	14.9 ± 0.5	9	12.3 ± 1.2	24	82.6 ± 8.7
Rb	7.39 ± 0.19	9	6.83 ± 0.54	24	92.4 ± 7.7
Cu	4.93 ± 0.15	9	4.99 ± 0.42	24	101.3 ± 9.1
Cr	4.27 ± 0.77	9	2.91 ± 0.48	7	68.2 ± 21.2
Se	0.582 ± 0.042	9	<LOQ	-	---
Ni	1.437 ± 0.242	7	1.264 ± 0.089	4	88 ± 18

Table S6 Determined mass fractions *w* and number of quantifications *n* in maternal tissue after a fixation time of 24 h after digestion (Dig) and suspension (Sus). Results are expressed as mean ± SD with N = 4.

Element	w_{Dig} / $\mu g\ g^{-1}$	*n*	w_{Sus} / $\mu g\ g^{-1}$	*n*	recovery / %
Ca	16,655 ± 305	9	17,210 ± 666	27	103.3 ± 4.4
K	6,551 ± 68	9	6,604 ± 623	27	100.8 ± 8.1
Fe	769 ± 10	9	719 ± 34	27	93.5 ± 4.6
Zn	61 ± 0.6	9	52.6 ± 0.8	27	86.2 ± 1.6
Sr	9.4 ± 0.3	9	8.5 ± 0.7	27	90.4 ± 8.1
Rb	10.3 ± 0.33	9	8.79 ± 0.51	27	85.3 ± 5.9
Cu	5.06 ± 0.09	9	4.62 ± 0.35	27	91.3 ± 7.2
Cr	3.71 ± 0.42	8	<LOQ	0	---
Se	0.58 ± 0.045	9	0.585 ± 0.027	5	100.8 ± 9.0
Ni	<LOQ	0	<LOQ	0	---

Table S7 Determined mass fractions w and number of quantifications n in fetal tissue after a fixation time of 36 h after digestion (Dig) and suspension (Sus). Results are expressed as mean ± SD with N = 3.

Element	w_{Dig} / $\mu g\ g^{-1}$	*n*	w_{Sus} / $\mu g\ g^{-1}$	*n*	recovery / %
Ca	34,575 ± 951	9	31,851 ± 3,094	18	92.1 ± 9.4
K	4,186 ± 42	9	4,462 ± 310	18	106.6 ± 7.5
Fe	560 ± 26	9	521 ± 31	18	93.0 ± 7.2
Zn	80.4 ± 4.1	9	67.1 ± 4.2	18	83.5 ± 7.3
Sr	20.9 ± 2.2	9	16 ± 1.8	18	76.6 ± 13.6
Rb	6.52 ± 1.01	9	5.57 ± 0.44	18	85.4 ± 16.9

Cu	4.82 ± 0.48	9	4.26 ± 0.28	18	88.4 ± 11.5
Cr	4.77 ± 1.07	7	3.84 ± 0.96	3	80.6 ± 30.2
Se	0.613 ± 0.074	5	<LOQ	0	---
Ni	2.759 ± 1.014	6	2.054 ± 0.379	15	74.4 ± 39.2

Table S8 Determined mass fractions *w* and number of quantifications *n* in intermediate tissue after a fixation time of 36 h after digestion (Dig) and suspension (Sus). Results are expressed as mean ± SD with N = 3.

Element	w_{Dig} / $\mu g\ g^{-1}$	*n*	w_{Sus} / $\mu g\ g^{-1}$	*n*	recovery / %
Ca	26,636 ± 1,319	9	27,317 ± 278	18	102.6 ± 5.1
K	5,342 ± 146	9	5,940 ± 399	18	111.2 ± 8.0
Fe	607 ± 11	9	590 ± 13	18	97.2 ± 2.8
Zn	76.5 ± 1	9	65.3 ± 1.2	18	85.4 ± 2.0
Sr	15.3 ± 0.8	9	13.3 ± 0.7	18	86.9 ± 6.9
Rb	8.18 ± 0.6	9	7.26 ± 0.54	18	88.8 ± 9.9
Cu	4.57 ± 0.36	9	4.08 ± 0.22	18	89.3 ± 9.3
Cr	4.02 ± 0.62	8	<LOQ	0	---
Se	0.594 ± 0.047	9	0.692 ± 0.083	3	116.6 ± 16.1
Ni	1.603 ± 0.35	6	1.474 ± 0.214	4	92 ± 25.6

Table S9 Determined mass fractions *w* and number of quantifications *n* in maternal tissue after a fixation time of 36 h after digestion (Dig) and suspension (Sus). Results are expressed as mean ± SD with N = 4.

Element	w_{Dig} / $\mu g\ g^{-1}$	*n*	w_{Sus} / $\mu g\ g^{-1}$	*n*	recovery / %
Ca	30,907 ± 878	9	28,593 ± 899	27	92.5 ± 4.1
K	4,979 ± 86	9	5,546 ± 299	27	111.4 ± 6.2
Fe	687 ± 20	9	624 ± 35	27	90.8 ± 5.9
Zn	83.9 ± 5.1	9	62.8 ± 1.4	27	74.9 ± 6.3
Sr	19.2 ± 1.8	9	13.7 ± 0.8	27	71.4 ± 10.3
Rb	8.54 ± 0.67	9	7.03 ± 0.47	27	82.3 ± 9.6
Cu	4.76 ± 0.4	9	3.98 ± 0.34	27	83.6 ± 11
Cr	5.62 ± 1.14	9	3.4 ± 0.83	6	60.4 ± 25.1

Se	0.711 ± 0.086	7	0.662 ± 0.102	4	93.1 ± 18.7
Ni	1.574 ± 0.125	3	1.727 ± 0.272	10	109.7 ± 19
